# Utilization of point-of-care ultrasound and associated factors among emergency physicians in Henan Province, China: a multicenter cross-sectional survey

**DOI:** 10.3389/fpubh.2026.1776608

**Published:** 2026-02-12

**Authors:** Yanwei Cheng, Zhaoyu Wang, Qi Zou

**Affiliations:** Department of Emergency, Henan Provincial People’s Hospital, People’s Hospital of Zhengzhou University, People’s Hospital of Henan University, Zhengzhou, China

**Keywords:** associated factors, cross-sectional survey, emergency medicine, point-of-care ultrasound, utilization

## Abstract

**Background:**

Real-world evidence on point-of-care ultrasound (POCUS) utilization and its correlates among emergency physicians remains limited in China. This study assessed POCUS use, practice patterns, training and perceived competence in Henan Province and examined physician- and hospital-level factors associated with POCUS utilization.

**Methods:**

A multicenter cross-sectional questionnaire survey was conducted in Henan Province from 1 October to 15 November 2025 among emergency physicians with at least 3 years of ED experience from a targeted network of 278 public secondary and tertiary general hospitals. We compared characteristics between POCUS users and non-users and used logistic regression to identify factors associated with POCUS use, with sensitivity analyses further adjusting for hospital bed size and annual emergency department volume.

**Results:**

Of 1,020 physicians who accessed the survey, 2 did not provide informed consent, leaving 1,018 respondents for analysis. Overall, 569 physicians (55.9%) reported any current POCUS use and 449 (44.1%) reported no POCUS use. Among non-users, the leading barriers were lack of an ultrasound machine (300, 66.8%) and lack of POCUS skills (160, 35.6%). Among respondents reporting POCUS use, the most common applications were basic procedural guidance (453, 79.6%) and FAST or extended FAST (423, 74.4%). Self-rated competence was modest, with 260 (45.7%) rating skills as fair and 201 (35.4%) as poor or very poor. Use during cardiac arrest was uncommon: 84 (14.8%) never used POCUS and 136 (23.9%) used it often or always. In the primary multivariable model, educational level (adjusted OR 2.06 per one-category increase, 95% CI 1.52–2.79), tertiary hospital status (adjusted OR 1.60, 95% CI 1.16–2.20), and teaching hospital status (adjusted OR 2.03, 95% CI 1.54–2.69) were associated with POCUS use. In sensitivity analyses, hospital bed size and emergency department volume were strongly associated with POCUS use, and tertiary hospital status was no longer statistically significant.

**Conclusion:**

POCUS was commonly reported among emergency physicians in Henan Province, but adoption was constrained by equipment and training gaps, with particularly low use during cardiac arrest. Institutional capacity appears to be an important correlate of POCUS uptake, supporting combined strategies of equipment provision and scalable competency-based training.

## Introduction

Point-of-care ultrasound (POCUS) has become an important diagnostic and monitoring modality in the management of critically ill and injured patients (1). Performed and interpreted at the bedside by the treating physician, POCUS provides rapid assessment of hemodynamics and organ pathology and can improve diagnostic accuracy, clinical decision-making, and procedural safety across a wide range of emergency presentations (2). In emergency medicine, POCUS is most commonly used in the form of focused assessment with sonography for trauma (FAST) or extended FAST in trauma, lung ultrasound in acute respiratory failure, and focused cardiac ultrasound during resuscitation (3–5). In Chinese EDs, trauma and injury-related complaints constitute a substantial proportion of visits (around one-third in some hospitals), which further underscores the clinical relevance of FAST and extended FAST in routine practice (6).

Despite these advantages, real-world POCUS utilization by emergency and critical care physicians remains highly variable. Surveys from high-income countries show that many emergency departments (EDs) still do not have mature POCUS programs and that use is strongly influenced by institutional context and physician training (7–10). In some national and regional surveys, only a minority of EDs report routine use of physician-performed POCUS, with substantially higher adoption in academic than in community hospitals and with marked variation in indications, training requirements and quality-assurance processes (9, 10). Multispecialty critical care surveys similarly indicate that most clinicians report at least occasional use of cardiac, lung or abdominal POCUS, but only a subset use these applications routinely in key scenarios such as undifferentiated hypotension or in-hospital cardiac arrest (11, 12). Across these studies, limited training opportunities, lack of competency and the absence of structured quality-assurance systems are repeatedly identified as major barriers to broader POCUS utilization.

In China, interest in POCUS within emergency and critical care practice has expanded rapidly, and national expert groups have begun to promote standardized training, competency-based curricula and quality-control frameworks for bedside ultrasound (13). However, China’s tiered healthcare system may introduce additional structural disparities in POCUS utilization (14). Tertiary teaching hospitals typically have greater access to ultrasound equipment, subspecialty expertise and formal training opportunities. In contrast, secondary and non-teaching hospitals, particularly those in less developed regions, may face constraints related to staffing, equipment and educational resources. Existing Chinese studies have largely focused on single centers, specific specialties such as intensive care, anesthesiology or prehospital care, or individual applications such as FAST or focused cardiac ultrasound, and have emphasized technical performance rather than patterns of use in routine emergency care. Consequently, comprehensive multicenter data describing how often POCUS is used by emergency physicians, in which clinical scenarios it is applied and how physician- and hospital-level factors influence its utilization remain limited.

Against this backdrop, we conducted a multicenter cross-sectional questionnaire survey of emergency physicians in Henan Province, China. Our objectives were to describe how frequently and in which clinical scenarios POCUS is used, to summarize POCUS-related training and self-perceived competence, and to examine physician- and hospital-level characteristics associated with POCUS utilization. By clarifying patterns and factors associated with POCUS use in this large provincial sample, this study aims to provide empirical evidence to inform future training strategies, equipment planning and policy development for emergency POCUS in China.

## Methods

### Study design and participants

This was a multicenter cross-sectional questionnaire survey conducted among emergency physicians in Henan Province, China. Data were collected from 1 October to 15 November 2025. The survey was disseminated through a targeted network of 278 public secondary and tertiary general hospitals that had at least 500 inpatient beds and a functionally independent ED. These hospitals included both teaching and non-teaching institutions and represented a range of hospital sizes and annual ED patient volumes within the province. Teaching status was self-reported and was defined as having a formal affiliation for undergraduate or postgraduate medical education (training of medical students and/or residents).

Eligible participants were physicians currently working in the ED of a targeted hospital with at least 3 years of clinical experience in emergency medicine. Non-physician staff, physicians from other departments and those with less than 3 years of ED experience were not invited. The study was reviewed by the Ethics Committee of Henan Provincial People’s Hospital, which waived the requirement for formal ethical approval. Participation was voluntary, and electronic informed consent was obtained from all respondents on the first page of the questionnaire.

### Data collection, recruitment and survey distribution

Data were collected using a structured, self-administered electronic questionnaire distributed via Questionnaire Star and completed through WeChat. The online platform required completion of all questionnaire items before submission, and partially completed questionnaires could not be submitted. At each targeted hospital, a local ED coordinator disseminated the survey link to eligible physicians through departmental WeChat groups. Coordinators were instructed to forward the link to all eligible ED physicians. However, hospital-level staffing counts and individual invitations were not tracked. As a result, the total number of physicians invited and the physician-level response rate could not be reliably determined. Participation was voluntary and anonymous, and physician-level recruitment did not use probability-based sampling. “Accessed” was defined as reaching the survey landing page and submitting the consent item, which was required to proceed. Responses were reviewed for consent and completeness before analysis. Hospital identifiers were not collected to preserve respondent anonymity.

### Duplicate prevention and data quality control

To reduce duplicate submissions, the online survey platform was configured to allow only one completed questionnaire per IP address. Based on this restriction, no duplicate records were identified for removal.

### Questionnaire development and content

The questionnaire was adapted from a previously published survey of bedside ultrasound use among emergency physicians by Stein et al. (7) and was modified to fit the Chinese ED context and the objectives of this study. Items on overall POCUS use, specific clinical applications (e.g., FAST/eFAST, biliary, cardiac, aortic, renal, pregnancy-related and procedural ultrasound), ED-level equipment and expectations regarding future ultrasound use were informed by the original instrument, with wording adjusted from the department level to the individual physician level. Additional items were newly developed to capture physician-level characteristics (age, sex, educational level, professional title), detailed hospital characteristics (hospital grade, teaching status, bed size, annual ED volume), POCUS-related training exposure, self-rated POCUS skill level, perceived impact of POCUS on clinical decision-making and barriers to use, as well as the use of POCUS during cardiac arrest and reasons for non-use.

To improve clarity and face validity, the draft questionnaire was reviewed internally by emergency physicians in our department and underwent a small-scale pretest with 20 physicians. Participants were asked to comment on item wording, response options, and overall comprehensibility. Based on their feedback, we simplified several item stems, merged or removed overlapping response categories and added “other” options with free-text fields where appropriate.

The final questionnaire consisted mainly of closed-ended single-choice, multiple-choice, and Likert-scale items and covered four domains: (1) physician and hospital characteristics; (2) overall POCUS use and reasons for non-use, as well as perceived future trends; (3) POCUS applications and training experiences among users; and (4) self-rated POCUS skills, perceived impact, and ED equipment. The full questionnaire is provided in Supplementary material S1. For non-users, barriers included a response option “no ultrasound machine in the ED,” whereas among users we additionally asked whether a dedicated POCUS machine was available in the ED. Therefore, ED equipment availability was not measured in a fully symmetric way across users and non-users. The primary outcome was any self-reported current use of POCUS in emergency practice, defined by the yes/no item “Do you use POCUS in your clinical practice”? Respondents who answered “yes” were classified as POCUS users, regardless of how frequently they used POCUS. Self-rated POCUS skill level and perceived impact on clinical decision-making were treated as subjective assessments of the respondents’ own abilities and practice rather than as objective measures of procedural competence or patient outcomes.

### Data analysis

Continuous variables were summarized as mean ± standard deviation (SD) or median with interquartile range (IQR), as appropriate. Categorical variables were expressed as counts and percentages. Differences between POCUS users and non-users were assessed using the Student’s *t* test or the Mann–Whitney U test for continuous variables and the *χ*^2^ test or Fisher’s exact test for categorical variables. For multiple-response items (such as reasons for not using POCUS, types of POCUS applications and training sources), each option was analyzed as a separate binary variable, and percentages were calculated using the corresponding subgroup as the denominator.

To explore factors associated with POCUS use, univariable logistic regression analyses were first performed with POCUS use as the dependent variable and each physician- or hospital-level characteristic as the independent variable. A multivariable logistic regression model then included age, sex, educational level, professional title, hospital grade and teaching status, with educational level and professional title entered as ordinal variables to reflect the ordered nature of these categories. This specification assumes an approximately linear trend in the log-odds of POCUS use across adjacent categories and may not capture more complex non-linear relationships. Because hospital grade, hospital bed size and annual ED volume all reflect institutional capacity and case throughput and bed size and ED volume may lie on the pathway between hospital grade and POCUS adoption rather than acting purely as confounders, we treated the model including hospital grade and teaching status as our primary specification. As a sensitivity analysis, we fitted an additional multivariable logistic regression model further adjusting for hospital bed size and annual ED patient volume. Results are presented as odds ratios (ORs) with 95% confidence intervals (CIs). All statistical analyses were conducted using IBM SPSS Statistics. A two-sided *p* value <0.05 was considered statistically significant, and analyses were based on complete cases. Because the online platform required completion of all questionnaire items before submission, there were no missing data for analysis variables among respondents who provided consent.

## Results

### Participant characteristics

Of eligible emergency physicians working in the 278 targeted hospitals, the exact number invited could not be determined because hospital-level staffing counts and individual invitations were not tracked. Overall, 1,020 physicians reached the online survey landing page and consent item, of whom 1,018 provided electronic consent and were included in the analysis. Median completion time was 116.5 s, with an interquartile range of 67 to 183 s. Among all respondents, 569 (55.9%) reported any current use of POCUS in their clinical practice and 449 (44.1%) reported not using POCUS.

Baseline characteristics by reported POCUS use are shown in Table 1. Compared with non-users, respondents reporting POCUS use were slightly older (38.3 ± 7.6 vs. 37.3 ± 7.5 years, *p* = 0.033) and had higher educational attainment (*p* < 0.001). They were also more likely to work in tertiary and teaching hospitals and in hospitals with larger bed capacity and higher annual ED patient volume (all *p* < 0.001). Sex distribution did not differ between groups (*p* = 0.95).

**Table 1:** Baseline characteristics by reported POCUS use.

Characteristic	Total (*N* = 1,018)	Non-users (*n* = 449)	POCUS users (n = 569)	*p* value
Age, years, mean ± SD	37.8 ± 7.6	37.3 ± 7.5	38.3 ± 7.6	0.033
Sex, *n* (%)				0.95
Male	628 (61.7)	276 (61.5)	352 (61.9)	
Female	390 (38.3)	173 (38.5)	217 (38.1)	
Highest educational level, *n* (%)				<0.001
Junior college / associate degree	51 (5.0)	25 (5.6)	26 (4.6)	
Bachelor’s degree	750 (73.7)	380 (84.6)	370 (65.0)	
Master’s degree	207 (20.3)	44 (9.8)	163 (28.6)	
Doctoral degree	10 (1.0)	0 (0.0)	10 (1.8)	
Professional title, *n* (%)				0.002
Resident physician	297 (29.2)	145 (32.3)	152 (26.7)	
Attending physician	496 (48.7)	230 (51.2)	266 (46.7)	
Associate chief physician	185 (18.2)	61 (13.6)	124 (21.8)	
Chief physician	40 (3.9)	13 (2.9)	27 (4.7)	
Hospital type, *n* (%)				<0.001
Tertiary teaching hospital	503 (49.4)	160 (35.6)	343 (60.3)	
Tertiary non-teaching hospital	257 (25.2)	131 (29.2)	126 (22.1)	
Secondary teaching hospital	72 (7.1)	33 (7.3)	39 (6.9)	
Secondary non-teaching hospital	186 (18.3)	125 (27.8)	61 (10.7)	
Number of hospital beds, *n* (%)				<0.001
500–999 beds	522 (51.3)	306 (68.2)	216 (38.0)	
1,000–1,999 beds	298 (29.3)	116 (25.8)	182 (32.0)	
≥ 2,000 beds	198 (19.4)	27 (6.0)	171 (30.1)	
Annual emergency department visits, last year, *n* (%)				<0.001
< 50,000 visits	546 (53.6)	322 (71.7)	224 (39.4)	
50,000–99,999 visits	307 (30.2)	106 (23.6)	201 (35.3)	
100,000–199,999 visits	114 (11.2)	16 (3.6)	98 (17.2)	
≥ 200,000 visits	51 (5.0)	5 (1.1)	46 (8.1)	

### Reasons for not using POCUS and perceived future trends

As shown in Table 2, the most common barrier among non-users was lack of an ultrasound machine in the ED, reported by 300 physicians (66.8%). The second most common reason was lack of POCUS skills, reported by 160 physicians (35.6%). Other reasons were reported less frequently. Most respondents expected POCUS use in their ED to increase over the next 1 to 3 years, reported by 803 physicians (78.9%), while 198 (19.4%) expected no major change and 17 (1.7%) expected a decrease.

**Table 2:** Reasons for no POCUS use and perceived future trends.

Item	*n* (%)
POCUS utilization among all physicians, *n* (%), *N* = 1,018	
Using POCUS	569 (55.9)
Not using POCUS	449 (44.1)
Main reasons for not using POCUS, *n* (% of non-users)ᵃ	
Do not know how to use POCUS	160 (35.6)
Lack of time	29 (6.5)
Prefer to use other imaging modalities	34 (7.6)
Do not trust POCUS results performed by non-sonographers	31 (6.9)
No ultrasound machine available in the ED	300 (66.8)
Other reasons	49 (10.9)
Perceived change in POCUS use in the ED over the next 1–3 years, *n* (%), *N* = 1,018	
Increase	803 (78.9)
Remain about the same	198 (19.4)
Decrease	17 (1.7)

ᵃMultiple responses were allowed. Percentages are calculated using the number of physicians who did not use POCUS (*n* = 449) as the denominator.

### POCUS practice patterns among POCUS users

Among respondents reporting POCUS use, the most commonly reported applications were basic procedural guidance and FAST or extended FAST, reported by 453 physicians (79.6%) and 423 (74.4%), respectively (Table 3). Nearly half reported performing advanced POCUS applications, reported by 272 physicians (47.8%). Use of POCUS during cardiac arrest was not routine. A total of 84 physicians (14.8%) reported never using POCUS in this setting, while 136 (23.9%) reported using it often or always. When used during resuscitation, identification of reversible causes was the most commonly reported purpose, reported by 412 physicians (72.4%). Departmental POCUS courses were the most frequently reported training source, reported by 340 physicians (59.8%). Self-assessed competence was modest overall, with 260 physicians (45.7%) rating their skills as fair and 201 (35.4%) as poor or very poor. Most respondents reporting POCUS use perceived a large or very large impact on clinical decision-making, reported by 353 physicians (62.1%). Notably, 128 physicians (22.5%) reported having no dedicated POCUS machine in their ED.

**Table 3:** POCUS applications, training, self-assessment and equipment among respondents reporting POCUS use (*N* = 569).

Item	POCUS users (*n* = 569)
POCUS applications, *n* (% of users)	
FAST/eFAST	423 (74.4)
Hepatobiliary ultrasound	340 (59.8)
Cardiac ultrasound	405 (71.2)
Abdominal aorta ultrasound	290 (51.0)
Renal ultrasound	339 (59.6)
Pregnancy-related ultrasound	210 (36.9)
Basic procedural guidance ultrasoundᵃ	453 (79.6)
Advanced POCUS applicationsᵇ	272 (47.8)
Use of POCUS for cardiac arrest, *n* (% of users)	
Never	84 (14.8)
Rarely	208 (36.6)
Sometimes	141 (24.8)
Often	108 (19.0)
Always	28 (4.9)
POCUS applications used during cardiac arrestᶜ, *n* (% of users)	
Identification of reversible causes	412 (72.4)
Cardiac standstill assessment	344 (60.5)
Assessment of CPR quality	292 (51.3)
Confirmation of endotracheal tube position	201 (35.3)
Other applications	88 (15.5)
Main training sourcesᵈ, *n* (% of users)	
Critical care fellowship/standardized critical care training	294 (51.7)
Residency training	163 (28.6)
Departmental POCUS course	340 (59.8)
Self-directed learning	318 (55.9)
Ultrasound subspecialty/fellowship training	158 (27.8)
Other training sources	72 (12.7)
Self-assessment of POCUS skills, *n* (% of users)	
Very poor	51 (9.0)
Poor	150 (26.4)
Fair	260 (45.7)
Good	89 (15.6)
Very good	19 (3.3)
Perceived impact of POCUS on clinical decision-making, *n* (% of users)	
Very small	23 (4.0)
Small	48 (8.4)
Neither small nor large	145 (25.5)
Large	252 (44.3)
Very large	101 (17.8)
Number of dedicated POCUS machines in the ED, *n* (% of users)	
0 devices	128 (22.5)
1 device	282 (49.6)
2 devices	80 (14.1)
3–5 devices	79 (13.9)

𝐻asic procedural guidance ultrasound includes vascular access, pericardiocentesis, abdominal or pleural drainage, foreign body localization, and abscess drainage. 𝑺dvanced POCUS applications include lower-limb DVT assessment, ocular and testicular ultrasound, pneumothorax assessment, Doppler applications, suprapubic bladder puncture, etc. ᶜMultiple responses were allowed; percentages are calculated using the total number of POCUS users (*N* = 569) as the denominator. ᵈMultiple training sources could be selected; percentages are calculated using the total number of POCUS users (*N* = 569) as the denominator.

### Factors associated with POCUS use

Table 4 summarizes the multivariable logistic regression results. After adjustment for age, sex, educational level, professional title, hospital grade, and teaching status, higher educational level was independently associated with POCUS use, with an adjusted OR of 2.06 (95% CI 1.52–2.79, *p* < 0.001) per one-category increase. Working in a tertiary hospital and in a teaching hospital were also associated with higher odds of POCUS use, with adjusted ORs of 1.60 (95% CI 1.16–2.20, *p* = 0.004) and 2.03 (95% CI 1.54–2.69, *p* < 0.001), respectively. Age showed a borderline association in the primary model (*p* = 0.052), while sex and professional title were not independently associated with POCUS use. In sensitivity analyses that additionally adjusted for hospital bed size and annual ED patient volume, hospitals with at least 2,000 beds and EDs with at least 100,000 annual visits were strongly associated with POCUS use, while tertiary hospital status was no longer statistically significant, with an adjusted OR of 1.07 (95% CI 0.75–1.54, *p* = 0.698), and age became statistically significant (*p* = 0.028; Supplementary Table S1).

**Table 4:** Univariate and multivariable logistic regression analysis of factors associated with POCUS use (*N* = 1,018).

Variable	Crude OR (95% CI)	*p* value	Adjusted OR (95% CI)	*p* value
Age, per 1-year increase	1.02 (1.00–1.04)	0.033	1.02 (1.00–1.05)	0.052
Male sex (*vs.* female)	1.02 (0.79–1.32)	0.877	1.16 (0.88–1.53)	0.297
Educational level, per 1-category increaseᵃ	2.66 (2.01–3.51)	<0.001	2.06 (1.52–2.79)	<0.001
Professional title, per 1-category increaseᵇ	1.32 (1.12–1.55)	<0.001	0.98 (0.78–1.24)	0.881
Tertiary hospital (*vs.* secondary)	2.52 (1.88–3.37)	<0.001	1.60 (1.16–2.20)	0.004
Teaching hospital (*vs.* non-teaching)	2.68 (2.08–3.47)	<0.001	2.03 (1.54–2.69)	<0.001

𝐾ducational level was coded as: junior college/associate degree = 1, bachelor’s degree = 2, master’s degree = 3, doctoral degree = 4. ᵇProfessional title was coded as: resident physician = 1, attending physician = 2, associate chief physician = 3, chief physician = 4.

## Discussion

In this multicenter cross-sectional survey of emergency physicians in Henan Province, more than half of respondents reported any current use of POCUS in their clinical practice, yet 44.1% reported not using POCUS at all. Among users, procedural guidance and FAST or extended FAST were the most common applications, whereas routine use during cardiac arrest was uncommon and self-rated competence was modest; among non-users, lack of an ultrasound machine in the ED and lack of skills were the leading reported barriers. In multivariable logistic regression analyses, higher educational level and working in tertiary and teaching hospitals were independently associated with POCUS use, and in a sensitivity model that additionally adjusted for hospital bed size and annual ED volume, the association with tertiary status was attenuated while larger bed size and higher ED volume remained strongly associated with POCUS use.

Our findings are consistent with international survey literature showing that POCUS diffusion is heterogeneous and closely tied to institutional capacity (7, 15). In a California ED survey, bedside ultrasound adoption differed markedly by institutional setting and was higher in academic environments than community sites, with variation in availability, credentialing, and quality assurance processes (7). These observations parallel our sensitivity analysis. In that analysis, hospital bed size and annual ED volume were strongly associated with POCUS use, whereas the association with tertiary hospital status was attenuated and no longer statistically significant. This pattern suggests that “hospital grade” may function partly as a proxy for resource capacity and clinical throughput rather than as an independent factor.

Barriers identified in our survey align with multispecialty critical care survey data indicating that lack of training, perceived competency gaps, and absence of structured quality support are common obstacles to broader uptake (15, 16). Because ED equipment data were not collected in the same way for all respondents and we did not independently verify machine availability, reports of “no ultrasound machine in the ED” should be interpreted as perceived lack of accessible equipment rather than a definitive absence of ultrasound capability. Taken together, these findings support an implementation strategy that combines equipment planning with scalable competency-based training and ongoing quality oversight (17). Conceptually, this is consistent with the broader view of POCUS as a clinician skill that requires longitudinal supervised practice and feedback rather than a one-time course. In lower-volume settings, such training could be delivered through a combination of brief focused courses, supervised scanning logbooks, periodic image review, and regional or tele-mentoring networks that allow clinicians to obtain feedback on recorded examinations. For cardiac arrest, practical approaches include predefining a POCUS operator within the resuscitation team, limiting image acquisition to brief pauses during rhythm checks, and using standardized protocols that specify when to defer ultrasound if views are inadequate or if image acquisition would prolong chest compression interruptions.

In our cohort, procedural guidance and FAST or extended FAST were the most frequently reported applications. This pattern is consistent with the long-standing role of FAST in trauma triage and the widespread use of ultrasound guidance for bedside procedures in emergency care. However, POCUS use during cardiac arrest was not routine. This finding is consistent with data from intensive care settings, where POCUS is often used selectively during cardiac arrest rather than as a standard component of every resuscitation. In a national survey of ICUs in the Department of Veterans Affairs, clinicians reported limited formal training, uncertainty about indications and how best to integrate POCUS into resuscitation, as well as concerns about interfering with chest compressions, as important barriers to more widespread use (12). In a prospective cohort study, use of POCUS during resuscitation was associated with prolonged pauses in cardiopulmonary resuscitation, reinforcing concerns that ultrasound may disrupt workflow if it is not carefully integrated into the arrest algorithm (18). These convergent findings across ED and ICU contexts suggest that increasing ultrasound use during resuscitation may require targeted training focused on rapid image acquisition and interpretation with strict attention to minimizing interruptions to chest compressions, together with local protocols that integrate POCUS into resuscitation roles and timing and clearly define when ultrasound should be deferred.

In the primary adjusted model, higher educational level and institutional factors were associated with POCUS use. In the sensitivity analyses, the association between tertiary hospital status and POCUS use was attenuated after additional adjustment for hospital size and ED volume, whereas larger hospital size and higher ED volume remained strongly associated with POCUS use. This pattern is plausible because larger hospitals and higher-volume EDs typically have greater access to equipment, more opportunities for supervised scanning and stronger educational infrastructure, and hospital grade, bed size and ED volume are conceptually overlapping and likely correlated. From a policy perspective, these findings suggest that closing the equipment gap and expanding structured training access in smaller or lower-volume settings may be more impactful than interventions anchored solely to hospital grade. Overall, because this study is cross-sectional and relies on self-reported measures, all regression results should be interpreted as associations rather than causal effects.

Age showed borderline significance in the primary model and became statistically significant in the sensitivity model, with a small effect size. This may reflect residual confounding by practice environment or differences in clinical experience not fully captured by professional title. Given the modest magnitude and threshold sensitivity, this finding should be interpreted cautiously and viewed as hypothesis-generating.

This study has several strengths, including a large physician sample across a wide range of public secondary and tertiary hospitals in a major province and the simultaneous examination of physician- and hospital-level characteristics. Limitations should also be considered. First, because the survey was disseminated by local coordinators and participation was voluntary, we could not determine how many eligible physicians actually received the link or calculate a physician-level response rate, so selection bias is possible and POCUS use may be overestimated. In addition, our sampling frame was restricted to public secondary and tertiary hospitals with at least 500 inpatient beds, and hospital identifiers were not collected, so we could not account for potential within-hospital clustering. These findings are therefore most directly applicable to mid- to large-sized public hospitals in Henan Province, and POCUS diffusion in smaller primary or rural hospitals is likely lower than suggested by our estimates. Second, eligibility was restricted to physicians with at least 3 years of emergency medicine experience, so junior physicians and residents were not represented and patterns of POCUS use and associations with age, educational level and professional title may differ in these groups. Third, all data were self-reported, and the primary outcome captured any current POCUS use rather than routine or time-bound use, while self-rated POCUS skill level and perceived impact on clinical decision-making reflect subjective perceptions rather than objective performance or clinical outcomes. In addition, we compared POCUS users and non-users across multiple physician- and hospital-level variables and reported percentages for numerous multi-response items without formal adjustment for multiple testing, so these exploratory comparisons should be interpreted with caution because some statistically significant differences may reflect chance. Fourth, ED equipment availability was not measured in the same way for all respondents and equipment was not independently verified, so responses about “no ultrasound machine in the ED” should be interpreted as perceived lack of access rather than definitive absence of ultrasound capability. Fifth, although the survey platform restricted submissions to one per IP address, this approach may not fully prevent duplicate participation and may also have excluded some eligible physicians who shared a workstation or network.

## Conclusion

In this large provincial survey of emergency physicians, POCUS use was common but unevenly implemented, with persistent barriers related to equipment and training and limited routine use during cardiac arrest. Institutional context and resource-related capacity appeared to be important correlates of POCUS uptake. These findings support targeted strategies combining equipment provision, competency-based training pathways, and programmatic quality support to expand effective POCUS integration in emergency care.

## Data Availability

The original contributions presented in the study are included in the article/Supplementary material, further inquiries can be directed to the corresponding author/s.
