# Supplementary material for: Utilization of point-of-care ultrasound and associated factors among emergency physicians in Henan Province, China: a multicenter cross-sectional survey
Source: Front Public Health. 2026 Feb 12;14:1776608. doi: 10.3389/fpubh.2026.1776608 (PMC12936038; doi:10.3389/fpubh.2026.1776608)
Supplement: Supplementary file 6 [file Table_6.DOCX]

### Supplementary Material S1

### Questionnaire on the use of point-of-care ultrasound (POCUS) by emergency physicians

Note: This is the English translation of the original Chinese questionnaire used in the study.

**Q1. Do you agree to participate in this study?** (Single choice)
☐ Yes
☐ No

Instruction: Respondents who select “No” exit the survey and no further data are collected.

**Q2. What is your age (years)?** (Free text)
____ years

**Q3. What is your sex?** (Single choice)
☐ Male
☐ Female

**Q4. What is your highest educational level?** (Single choice)
☐ Doctoral degree
☐ Master’s degree
☐ Bachelor’s degree
☐ Junior college / associate degree

**Q5. What is your professional title?** (Single choice)
☐ Chief physician
☐ Associate chief physician
☐ Attending physician
☐ Resident physician

**Q6. What is the type of your current hospital?** (Single choice)
☐ Tertiary teaching hospital
☐ Tertiary non-teaching hospital
☐ Secondary teaching hospital
☐ Secondary non-teaching hospital

**Q7. What is the total number of inpatient beds in your hospital?** (Single choice)
☐ 500–999
☐ 1,000–1,999
☐ ≥2,000

**Q8. What was the approximate number of ED visits to your hospital last year?** (Single choice)
☐ <50,000
☐ 50,000–99,999
☐ 100,000–199,999
☐ ≥200,000

**Q9. Do you use point-of-care ultrasound (POCUS) in your clinical practice?** (Single choice)
☐ Yes (→ skip to Q11)
☐ No (→ answer Q10, then submit the questionnaire)

**Q10. What are your main reasons for not using POCUS?** (Multiple choice)
☐ I do not know how to use POCUS.
☐ I do not have enough time.
☐ I prefer to use other diagnostic methods first.
☐ I do not trust POCUS results when performed by non-radiologists.
☐ I only treat patients who would not benefit from POCUS.
☐ There is no ultrasound machine in my department/ward.
☐ Other (please specify): _____________

Instruction: Respondents who select “No” in Q9 complete Q10 and then submit the questionnaire.

The following questions (Q11–Q21) are shown only to respondents who answered “Yes” to Q9.

**Q11. Through which pathways have you mainly received POCUS training?** (Multiple choice)
☐ Critical care medicine specialist/standardized training
☐ Standardized residency training
☐ Self-directed learning
☐ Ultrasound specialty/subspecialty training or fellowship
☐ In-department ultrasound training courses
☐ Other (please specify): _____________

**Q12. In your opinion, which of the following pathways is most effective for mastering POCUS skills?** (Single choice)
☐ Critical care medicine specialist/standardized training
☐ Standardized residency training
☐ Self-directed learning
☐ Ultrasound specialty/subspecialty training or fellowship
☐ In-department ultrasound training courses
☐ Other (please specify): _____________

**Q13. Which of the following POCUS applications can you currently perform in your clinical practice?**
Please indicate “Yes” or “No” for each item.

a. **FAST (including eFAST) for trauma**
 ☐ Yes  ☐ No

b. **Biliary ultrasound** (e.g. gallbladder, bile ducts)
 ☐ Yes  ☐ No

c. **Cardiac ultrasound** (focused cardiac POCUS)
 ☐ Yes  ☐ No

d. **Abdominal aorta ultrasound**
 ☐ Yes  ☐ No

e. **Renal ultrasound**
 ☐ Yes  ☐ No

f. **Pregnancy-related ultrasound**
 (e.g. screening for early intrauterine or ectopic pregnancy)
 ☐ Yes  ☐ No

g. **Basic procedure-guidance ultrasound**
 (e.g. vascular access, pericardiocentesis, paracentesis, thoracentesis, foreign-body localization, abscess drainage/localization, etc.)
 ☐ Yes  ☐ No

h. **Advanced ultrasound applications**
 (e.g. lower-limb deep vein thrombosis [DVT] assessment, ocular ultrasound, testicular ultrasound, pneumothorax assessment, Doppler applications, suprapubic bladder puncture, etc.)
 ☐ Yes  ☐ No

**Q14. How often do you use POCUS to evaluate patients with cardiac arrest?** (Single choice)
☐ Never
☐ Rarely
☐ Sometimes
☐ Often
☐ Always

**Q15. When you use POCUS in the evaluation of cardiac arrest, which applications do you perform?** (Multiple choice)
☐ Confirmation of endotracheal tube position
☐ Assessment of cardiopulmonary resuscitation (CPR) quality
☐ Identification of cardiac standstill
☐ Identification of potentially reversible causes
 (e.g. pericardial tamponade, tension pneumothorax, thrombosis/pulmonary embolism, hypovolemia, etc.)
☐ Other (please specify): _____________

**Q16. If you do not use POCUS to evaluate cardiac arrest, what are your main reasons?** (Multiple choice)
☐ Insufficient POCUS skills
☐ Insufficient equipment
☐ Serving as team leader / need to direct the resuscitation
☐ I believe POCUS has limited value in cardiac arrest
☐ Insufficient POCUS training
☐ Difficult to obtain adequate acoustic windows
☐ Other (please specify): _____________

**Q17. How would you rate your own skill level in performing emergency POCUS?** (Single choice)
☐ Very poor
☐ Poor
☐ Fair
☐ Good
☐ Very good

**Q18. To what extent do you think the POCUS examinations you perform influence clinical decision-making?** (Single choice)
☐ Very small
☐ Small
☐ Neither small nor large
☐ Large
☐ Very large

**Q19. Does your emergency department have its own POCUS equipment (dedicated ultrasound machine for POCUS in the ED)?** (Single choice)
☐ Yes
☐ No

**Q20. If yes, how many POCUS machines does your emergency department have?** (Single choice)
☐ 1 device
☐ 2 devices
☐ 3–5 devices

**Q21. How do you expect the use of POCUS in your emergency department to change over the next 1–3 years?** (Single choice)
☐ Increase
☐ Decrease
☐ Remain about the same
